# Foaming at the mouth: A case of psychogenic nonepileptic seizure

**DOI:** 10.1002/jgf2.70053

**Published:** 2025-10-17

**Authors:** Satoshi Saito, Go Taniguchi

**Affiliations:** ^1^ Department of Epileptology National Center Hospital, National Center of Neurology and Psychiatry Kodaira, Tokyo Japan; ^2^ Department of Neurology Tokyo Women's Medical University School of Medicine Tokyo Japan

**Keywords:** conversion disorder, dissociative disorders, epilepsy, incontinence, psychogenic non‐epileptic seizures

The patient is a 19‐year‐old female with a history of tonic–clonic seizures. Treatment with valproic acid (VPA) began at age 3 and was discontinued upon achieving seizure freedom at age 10. At age 14, she experienced maternal abuse and school difficulties, followed by episodes of collapse and unresponsiveness, sometimes with foaming at the mouth and small shaking, lasting up to an hour. She was diagnosed with a dissociative disorder at a psychiatric clinic. At age 17, she was admitted to the emergency room for a similar seizure and began treatment for epilepsy with levetiracetam and VPA. At age 19, she underwent video electroencephalography (VEEG) to address persistent intractable seizures.

After discontinuation of antiepileptic medications, VEEG was performed over 4 days. On day 2, a psychogenic nonepileptic seizure (PNES) was recorded. Although lying in bed and stating she felt “sick,” she abruptly exhibited bilateral shoulder twitching, unresponsiveness, and foaming at the mouth, but lacked tonic posturing, cyanosis, or oxygen desaturation, with her eyes remaining closed (Figure 1A). The EEG showed a normal posterior‐dominant rhythm (9–10 Hz) without epileptiform activity (Figure 1B). The physician examined her 5 min after seizure onset; she was unresponsive, exhibited strong resistance to forced eye‐opening, scored positive in arm‐drop and knee‐standing tests, and exhibited a bilateral flexor plantar response. After the physician exited the room, urinary incontinence occurred, and she gradually regained full consciousness.

**FIGURE 1:**
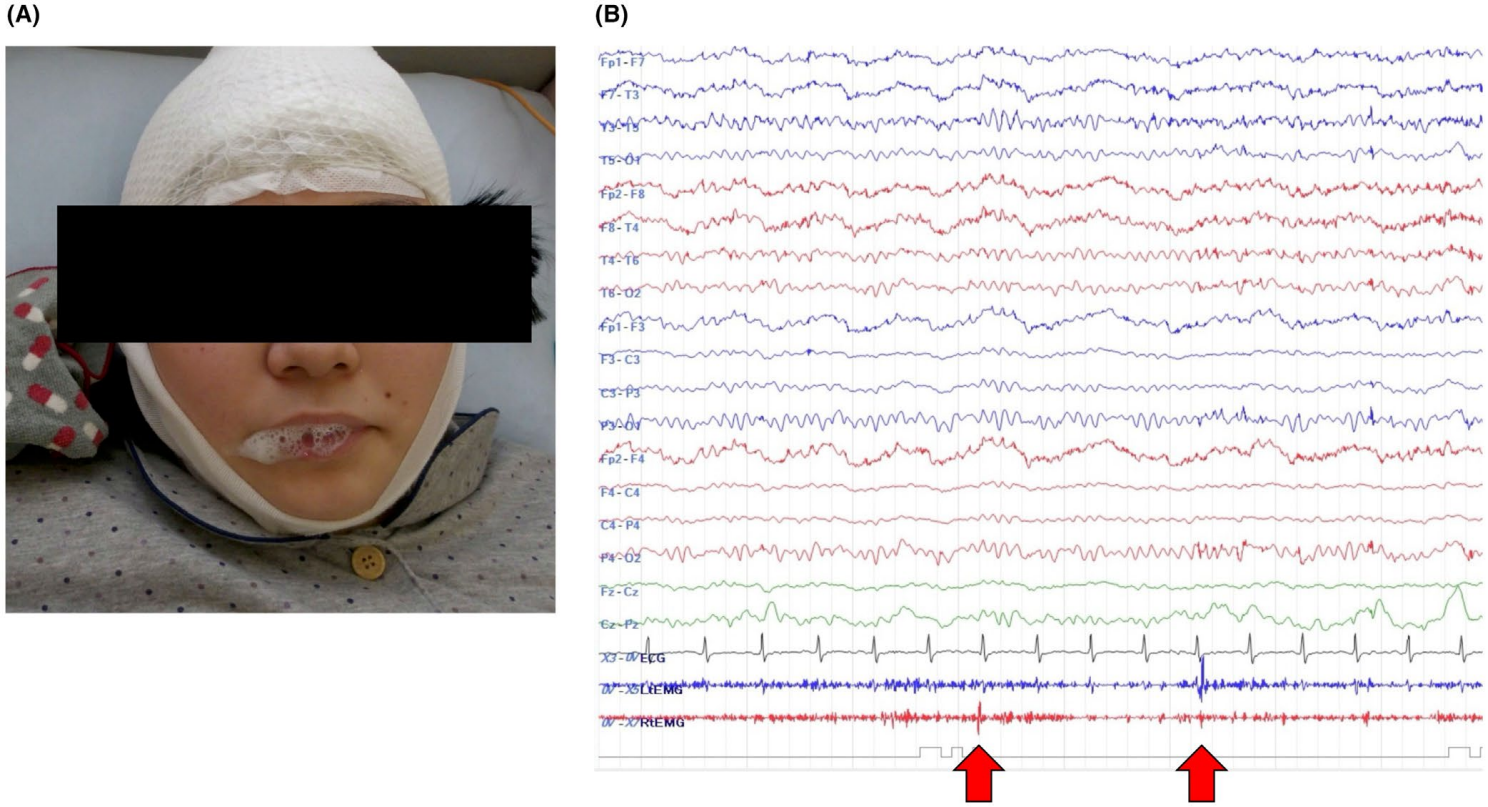
(A) Foaming at the mouth occurred after bilateral shoulder twitching and loss of responsiveness. (B) Electroencephalography (EEG) findings during foaming at the mouth and shoulder twitching. No epileptiform activity was observed and a normal posterior dominant rhythm (9–10 Hz) was maintained. The EEG conditions included a bipolar montage, a high‐cut filter of 120 Hz, and a time constant of 0.1 s. Red arrows indicate shoulder twitch activity from the deltoid muscles on surface electromyography (blue: Left, red: Right).

The patient's PNES likely stemmed from psychological stressors, such as maternal abuse and school difficulties. Addressing such underlying issues—through sustained psychiatric care, trauma‐informed therapy, and social support—may be crucial not only for reducing PNES frequency but also for achieving long‐term psychological stabilization.

PNESs have a prevalence of 2–33 per 100,000, whereas epilepsy affects approximately 0.5%–1% of the population.^1^ Unlike epilepsy, PNES are not caused by neuronal hypersynchrony or cerebral hypoperfusion but rather by complex neuropsychiatric mechanisms.^2^ Nonetheless, their semiology often mimics that of epileptic seizures, leading to misdiagnosis in 20%–30% of cases.^3^ Symptoms such as foaming at the mouth and urinary incontinence—observed in this case—are typically associated with generalized tonic–clonic seizures. In epilepsy, foaming is thought to result from hypersalivation and clonic breathing^3^; however, the mechanism behind this phenomenon in PNES remains unclear and warrants further investigation. This case contributes to the limited literature on such rare PNES manifestations and may serve as a reference for future research.

VEEG remains the gold standard for distinguishing PNES from epilepsy, although the likelihood of capturing an event during monitoring is only 50%–70%.^4^ Notably, a clear diagnosis alone can significantly reduce seizure recurrence and healthcare utilization. One study demonstrated that providing a definitive diagnosis prevented recurrence in 38% of patients and reduced annual emergency department visits from 49.7% to 15.5%.^5^ Therefore, in cases where distinguishing PNES from epilepsy is challenging—especially in presentations involving incontinence or foaming—prompt referral to an epilepsy specialist is crucial.

## AUTHOR CONTRIBUTIONS

**Satoshi Saito:** Conceptualization; writing – original draft; investigation. **Go Taniguchi:** Writing – review and editing.

## CONFLICT OF INTEREST STATEMENT

Authors declare no conflict of interests for this article.

## ETHICS STATEMENT

Ethics approval statement: None.

Patient consent statement: Written informed consent was obtained from the patient for publication.

Clinical trial registration: None.
